# Sleep and mental health among Chinese adolescents: the chain-mediating role of physical health perception and school adjustment

**DOI:** 10.1186/s40359-024-01719-4

**Published:** 2024-04-24

**Authors:** Liangliang Li, Yueying Zhang, Mingyue Fan, Bing Cao

**Affiliations:** 1 Chongqing Three Gorges Medical College, 366 Tianxing Road, 404120 Wanzhou, Chongqing, P. R. China; 2 Key Laboratory of Cognition and Personality, Faculty of Psychology, Ministry of Education, Southwest University, 400715 Chongqing, P. R. China; 3 National Demonstration Center for Experimental Psychology Education, Southwest University, 400715 Chongqing, P. R. China

**Keywords:** Sleep deficiency, Mental health, Perception of physical health, School adjustment, Adolescents

## Abstract

**Objective:**

Sleep problems and their detrimental effects on adolescents’ physical and mental health have received substantial attention. Prior studies have focused mainly on the direct association between sleep and mental health; however, little is known about the underlying mediating mechanism. To address this gap, the present study constructed a chain mediation model to examine the association between sleep deficiency and mental health status in adolescents, by introducing two mediating variables-physical health perception and school adjustment.

**Methods:**

A sample of 7530 senior high school students completed a battery of self-report questionnaires measuring their sleep duration, mental health status, physical health perception, and school adjustment. Data were collected from the Database of Youth Health at Shandong University. All the measures showed good reliability and validity in the present study. Data were analyzed using SPSS 25.0 and the SPSS PROCESS.

**Results:**

The results were as follows: (1) Sleep duration was significantly associated with physical health perception and mental health. (2) Physical health perception partially mediated the association between sleep and mental health. (3) Physical health perception and school adjustment played a chain mediating role between sleep and mental health. In conclusion, sleep not only directly associated with mental health among adolescents, but also influences mental health by the chain mediating effect of perception of physical health and school adjustment.

**Conclusion:**

These findings in the present study contribute to understanding the mechanisms underlying the association between sleep and mental health and have important implications for interventions aimed at improving mental health status among adolescents in China. Our results indicated that promoting adequate sleep duration and improving sleep quality are possible key mental health promotion strategies for adolescents.

**Supplementary Information:**

The online version contains supplementary material available at 10.1186/s40359-024-01719-4.

## Introduction

Sleep is a fundamental operating state of the central nervous system, consuming up to a third of the human life span. Recent and emerging data indicate a key role of sleep in supporting cognitive function and mental well-being [1]. Driven by delays in the circadian timing system and changes to the homeostatic sleep regulating system [2], bedtimes become later during adolescence. By contrast, rise times when adolescents wake up are more often determined by school start times and thus remain unchanged or move earlier [1]. Whether using self-report or objectively recorded sleep, studies showed that sleep restriction has become a main health problem among adolescents in China. The average school-night total sleep time for adolescents aged 15–17 years was about 7.9 h, and about 37%of them (13–17 years old) slept < 8 h per night, lower than the socially recommended 8–9 h [3]. Compared to a decade ago, the average sleep duration of Chinese adolescents has decreased [4, 5]. The trend of inadequate sleep and affected health problems represents a major public health concern [6, 7].

### Sleep and mental health

The detrimental impact of insufficient sleep has been found in adolescents targeting several domains: learning and memory, emotion regulation, behavioral problems, etc [8].., particularly prominent with mental health outcomes [9–11]. As a result of that, sleep deficiency is always associated with poor emotional functioning and symptoms of unfavorable mental health conditions, such as depressive symptoms [12, 13], and feelings of increased irritability and anxiety [14–16]. According to the evidence from animal models and/or pharmacological work, a plethora of neurotransmitter and neuromodulator systems orchestrates sleep and emotional regulation, including but not limited to norepinephrine, acetylcholine, γ-aminobutyric acid (GABA), dopamine, glutamate, serotonin [17, 18]. Moreover, sleep is reported to optimize the functioning of the prefrontal cortex (PFC), sleep deprivation could interfere the cognitive modulation of impulses, drives, and emotions governed by PFC [19, 20]. Therefore, in the current study we also speculate that sleep duration is significantly correlated with mental health. For all mental health conditions, depression and anxiety disorders are main mental problems, and confer a significant public health concern for youth and lead youth at a higher risk for poorer psychosocial outcomes [21, 22]. Meanwhile, we also conducted specialized discussions on the relationship between sleep restriction and depressive symptoms and anxiety, respectively.

### Mediating role of perception of physical health

Physical health perception refers to an individual’s perception of his or her current health [23]. It is generally used as a reliable and valid measure of assessing individuals’ subjective and objective health [24]. Extant evidence indicated that physical health perception significantly associated with mental health problems, such as insomnia, anxiety, depression, and distress, alongside a study conducted among the undergraduate found that students dissatisfied with their physical health status had higher changes from depression and anxiety [25]. Additionally, perceived health conditions could further influence social regular activities, for those who do not cope with the problem aggressively may show a social decline, like emotional unstable or difficulty with self-esteem or self-determination. Therefore, based on empirical evidence, we infer that the impact of sleep on mental health may be mediated by the perception of physical health.

### Mediating role of school adjustment

Adolescence is a time of increased independence and the emergence of new social roles. School adjustment refers to the ability to adapt to school, and suggests the interfusion of cognition, attitude, and behavior [26]. School adjustment includes variables such as academic performance, adaptation to school rules, respect for the teacher as an authority figure, a positive attitude toward school, and participation in school activities [27]. Studies have highlighted that better school adjustment has a positive relationship with mental health [28]. Thus, school adaption was also seen as significantly associated with mental health [29]. Therefore, based on this evidence, we can infer that sleep may influence mental health through school adjustment.

### Chain-mediating role of perception of physical health and school adjustment

To our knowledge, the relationship between perceived physical health and school adjustment and its mediating effects on sleep and mental health remains unknown. Numbers of existing studies indicated that poor and inadequate sleep negatively impacts cognitive and physical functioning, and sleep has a critical role in promoting health and health perception [30–32]. The existing evidence suggests school adjustment is a multi-causal construct that is manifested, it is associated with social, emotional, and physical aspects [33]. It was supported by a previous study that perceived physical health conditions and school adjustment is positively correlated [34]. Moreover, prior research has found that poor sleep has a negative influence on school adjustment and motivation for school activities [35].

Above all, the relationship between sleep and mental health overlaps due to the neurobiological centers of sleep and the neurobehavioral systems underlying arousal and emotions [36]. We propose a mechanism where during adolescence, the responsibility for individual judgment increases and the emergence of new social roles, while adult supervision gradually decreases over time, further affecting mental health through sleep affecting individual perceived physical health and poor school adaptation. Therefore, we inferred that both perceived physical health and school adjustment have a chain-mediating effect on the relationship between sleep and mental health. Based on exploring the relationship between sleep and overall mental health status, our study also aims to further explore the relationship between sleep and sub-dimensional psychological problems of depression and anxiety by adopting multivariate hierarchical regression analysis, as for which are prominent psychological problems in adolescents.

## Methods

### Subjects and procedure

Data were drawn from the Database of Youth Health (2021) from National Population Health Data Center (https://www.ncmi.cn/phda/dataDetails.do?id=CSTR:17970.11.A0031.202107.209.V1.0). The dataset provides information on Chinese youth on physical fitness, social interaction, nutrition and diet, school adaption, quality of life, psychological cognition, mental health, spare-time physical activity, and risk behavior. The dataset was a muti-wave survey conducted annually in the academic year of 2015/2016, 2016/2017, 2017/2018, and 2020/2021. A total of 99,327 students from 186 schools in all 17 cities of Shandong province participated in this survey [37].

In this study, we used the data of high school students’ information collated from the 2020/2021 semester. We merged the Nutritional & Dietary dataset, School Adaption dataset, and Mental Health dataset of subjects. Inclusion criteria were: the participants were high school students at the time of measurement. Participants who had missing values in sleep duration and perceived physical health status were excluded. It was well-noted by all participants that all data was collected voluntarily, anonymously, and confidentially, reserved on a password-protected website, and accessible only to direct researchers. Both parents and students completed informed consent forms before beginning this survey. This study has been approved by the Ethics Committee of Shandong University (20,180,517). Written informed consent was obtained from each participant.

### Measures

#### Sleep outcomes

In the current study, we focus on an important measure of sleep, sleep duration, which typically refers to the total amount of sleep obtained, either during the nocturnal sleep episode or across 24 h [38]. Participants were asked, “How many hours of sleep do you get on most nights?”. In this paper, we use “sleep duration” as the descriptor, where a high sleep duration indicates a large number of hours of sleep [39].

#### Perception of physical health

The participant’s perception of the condition of their physical health was assessed by using a one-item question covering information related to self-rated health. Students were asked to rate their physical health, “What do you think about your health status?”. The question is on a five-point scale (1 = very good, 2 = good, 3 = moderate, 4 = poor, and 5 = very poor) [25], with lower scores indicating higher subjective health levels.

#### School adjustment

School adjustment was measured by the School Social Behavior Scales (SSBS-2), which was originally developed by Merrell in 1993 for screening and assessing social competence and antisocial behavior of students from grades 1 to 12. The scale of SSBS-2 (Social Competence and Antisocial Behavior) consist of 65 items rated on a 5-point Likert-type scale. Each item was graded from 1 (never happen) to 5 (often happen), details are shown in Supplemental Table 1. A higher total score means better adaptive ability. In this study, Cronbach ɑ was 0.88.

#### Mental health

The self-assessment Symptom Checklist, also known as the Symptom Checklist 90 (SCL-90), was used to assess participants’ mental problems. The SCL-90 contains 90 self-assessment items, including 9 subscales of somatization, obsessive symptoms, interpersonal sensitivity, depression, anxiety, hostility, paranoia, and psychosis, which reflect psychological symptoms from the perspectives of feeling, emotion, thinking, consciousness, behavior, living habits, interpersonal relationship, diet, and sleep, respectively. Each item was evaluated on a 5-point scale (1 = never, 2 = mild, 3 = moderate, 4 = fairly severe, and 5 = severe), with a higher score indicating a severer level of psychological symptoms, details are shown in Supplemental Table 2. In this study, Cronbach ɑ was 0.94. In the analysis, we first used the total score to represent the overall mental health status, exploring the relationship between sleep and whole mental health condition. Based on that, we calculate the sub-dimensional scores of “depression” and “anxiety”, which are considered two common mental disorders in adolescents, and we further explored the relationship between sleep, depression & anxiety symptoms.

### Statistical analysis

SPSS25.0 and the PROCESS program complied by Hayes (2013) were used for data processing. We used sleep duration (hours) to represent sleep condition, adopted self-rated health to indicate perceived physical health, used scores of school adaption items, and SCL-90 to represent school adjustment and mental health status respectively. First, Pearson’s correlations were found to determine the associations between sleep, perception of physical health, school adjustment, and mental health. Hayes’ SPSS Process Macro Model 6 was employed to obtain quantitative evidence on the serial multiple mediation effects of physical health perception and school adjustment. Specifically, this model was used for evaluating mediated models of physical health perception and school adjustment, by adopting multivariate hierarchical regression analysis. A statistically significant *p*-value of 0.05 was used. The bootstrap confidence interval (CI) was set at 95%, and there were 5000 bootstrap samples.

## Results

### Sociodemographic characteristics of subjects

**Fig. 1:**
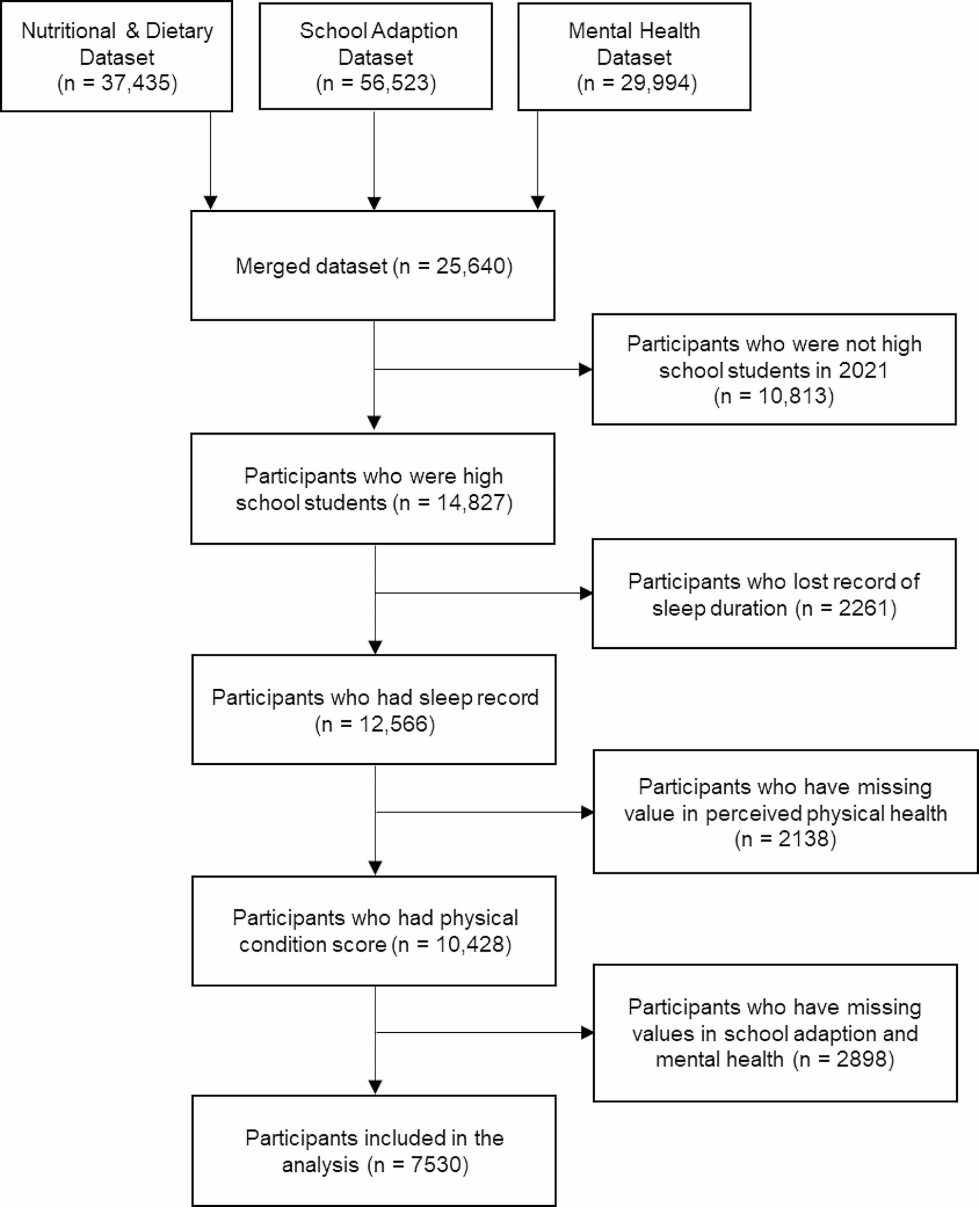
Inclusion process of participants

A total of 7530 participants were included in the analysis herein, after sorting out and deleting regular answers and missing questionnaires (Fig. 1). Frequency analysis was conducted to identify the general characteristics of study participants. According to the analysis, 4103 (54.5%) boys and 3427 (45.5%) girls were included in this study, and the age was from 15 to 18 years old (M = 17.21, SD = 2.36).

### Descriptive statistics and correlation analysis of variables

A correlation analysis between mental health, sleep duration, perception of physical health, and school adjustment is shown in Table 1. Sleep duration has a significant negative correlation with mental health problems (*r*=-0.196, *p* < 0.001), but a positive correlation with school adjustment (*r* = 0.031, *p* = 0.007). Physical health perception was significantly and positively associated with mental health (*r* = 0.343, *p* < 0.001). School adjustment was correlated with mental health problems negatively (*r*=-0.370, *p* < 0.001).

**Table 1:** Descriptive statistics and correlation coefficient of variables(*n* = 7530

Variables	M	SD	1	2	3	4
1 Mental health	1.49	0.56	1			
2 Sleep duration	6.97	1.09	-0.196^***^	1		
3 Physical health perception	2.51	1.06	0.343^***^	-0.249^***^	1	
4 School Adjustment	4.26	0.48	-0.370^***^	0.031^**^	-0.228^***^	1

Note. *p<0.05,**p<0.01,***p<0.001

*r* = 0.031 *p* = 0.007

### The relationship between sleep and mental health: chain mediation effect test

Regression analysis for evaluating mediated models of physical health perception and school adjustment is shown in Table 2. Insufficient sleep negatively associated with physical health perception (β=-0.249, t=-22.280, *p* < 0.001). Perceived physical health problems had a significant negative association with school adjustment (β=-0.235, t=-20.247, *p* < 0.001). Sleep duration (β=-0.065, t=-12.094, *p* < 0.001) and school adjustment (β=-0.362, t=-29.847, *p* < 0.001) were significant in negatively relationship with mental health problems, and physical health perception can positively link with mental health (β = 0.127, t = 22.370, *p* < 0.001).

**Table 2:** Regression analysis of the association between study variables

Variables	PPH			SA			MH		
	B	SE	t	B	SE	t	B	SE	t
Constant	4.210	0.077	54.628	4.607	0.041	111.391	3.169	0.071	44.692
SD	-0.249	0.011	-22.280^***^	-0.028	0.005	-2.380^*^	-0.065	0.005	-12.094^***^
PPH				-0.235	0.005	-20.247^***^	0.127	0.006	22.370^***^
SA							-0.362	0.012	-29.847^***^
*R* ^2^	0.062			0.053			0.223		
*F*	496.408^***^			208.730^***^			720.265^***^		

Note. Sleep duration = SD, Perception of physical health = PPH, School adjustment = SA, Mental health = MH. *p<0.05,**p<0.01,***p<0.001. For the association between SD and SA: t=-2.380, *p* = 0.017

**Fig. 2:**
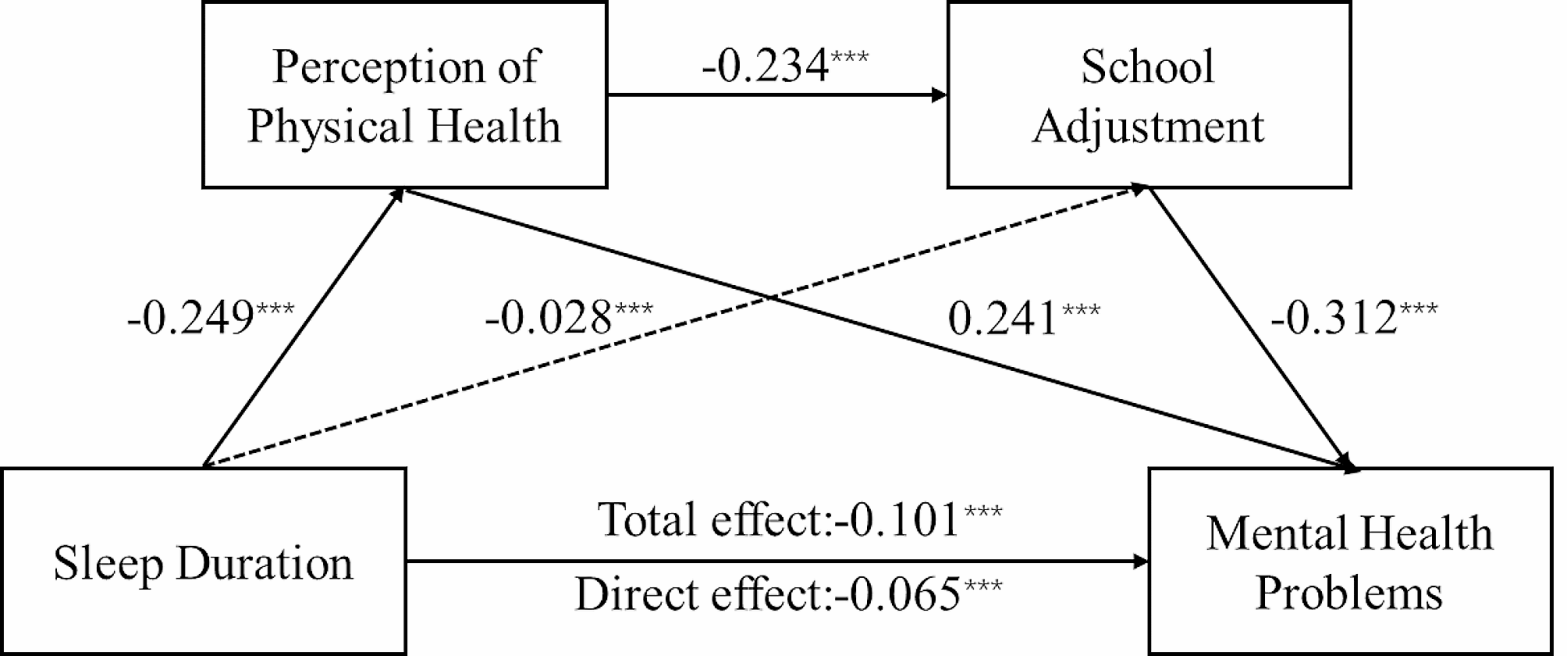
Diagram for the study variables association. ^***^p < 0.001

The non-parametric percentile Bootstrap method of bias correction provided by Hayes (2013) was used to test the mediation effect, and the 95% confidence interval was calculated for 5,000 repeated samples. If the confidence interval does not contain a value of 0, it means statistical significance. The results (Table 3) showed that: the bootstrap 95% confidence interval for the total indirect effect (-0.0358) of perceived physical health and school adjustment did not contain a value of 0, indicating the two mediators had a significant mediating effect between sleep and mental health. The total indirect effect is composed of three indirect effects: (1) indirect effects caused by sleep duration– perception of physical health– mental health (-0.0309); (2) indirect effects of sleep duration– perception of physical health– school adjustment– mental health (-0.0094); (3) indirect effects of sleep duration– school adjustment– mental health (0.0044). The indirect effect 3 is not significant for the confidence interval containing zero. In contrast, indirect effects generated by path 1 and path 2 reach significant levels, with a confidence interval not containing zero. Based on the above analysis, the chain mediation model is shown in Fig. 2.

**Table 3:** Mediating effect analysis of the chain mediating model

Items	Effect size	Boot SE	Boot CI		The Proportion of Effect Size
			Lower	Upper	
Total indirect effects	-0.036	0.004	-0.043	-0.029	35.41%
Indirect effect 1	-0.031	0.002	-0.036	-0.027	30.56%
Indirect effect 2	-0.009	0.001	-0.011	-0.008	9.30%
Indirect effect 3	0.004	0.003	-0.001	0.010	

Note. Indirect effect 1: sleep duration– perception of physical health– mental health; Indirect effect 2: sleep duration– perception of physical health– school adjustment– mental health; Indirect effect 3: sleep duration– school adjustment– mental health

Moreover, the results between sleep and depression & anxiety symptoms are consistent with the overall pattern of the above results. Sleep duration also significantly associated with depression and anxiety problems (β=-0.082, t=-15.066, *p* < 0.001; β=-0.074, t=-14.132, *p* < 0.001), and can have indirect effects through physical health perception and school adjustment. Specific results are shown in Supplemental Table 3.

## Discussion

In the present study, we focused on the mental health of adolescents who lack sleep. We sought to provide new evidence on the underpinning process and conceptualize mechanisms that can explain the negative link between sleep duration and adolescents’ mental health problems. The empirical evidence strongly supported our hypothesized chain mediation model, which highlights how insufficient sleep might affect adolescents’ mental health by reinforcing their perception of physical health and subsequently facilitating a successful school adjustment.

Sleep restriction negatively impacted adolescents’ mental health. Our findings are consistent with past studies that reported a negative link between sleep loss and mental health [40, 41]. One possible reason is that sufficient sleep, especially REM sleep, facilitates the brain’s processing of emotional information, and it appears that a lack of sleep is especially harmful to the consolidation of positive emotional content [42]. Subsequently, sleep loss can influence mood and emotional reactivity and is tied to mental health disorders and their severity, including the risk of suicidal ideas or behaviors [43]. Therefore, this research has significant ramifications for educational practices, for sleep is a modifiable behavior for many teens and effective interventions exist. At a basic level, the practice of good “sleep hygiene” protects sleep [44], such as limiting napping, establishing a nightly routine, avoiding caffeine or stimuli too close to bedtime, etc.

Furthermore, the findings of this study established the hypothesis validity of perceived physical health that played a mediating role between sleep and mental health problems. The effect of sleep restriction can be explained from two aspects: on the one hand, it has a physiological impact, such as the level of mental activity and attention concentration; on the other hand, it affects the cognitive beliefs about the assessment of the physical state, and negative beliefs will have an impact on emotions and behaviors, which effectively supported Dozois and Beck’s (2008) cognitive model of depression. Combined with the cognitive model, past studies confirmed that perception of physical health is related to mental health, such as poor self-rated health status had a significant association with depressive and anxiety symptoms among university students in some regions [45–47].

Additionally, a significant path of sleep duration– perception of physical health– school adjustment– mental health was found. The results between sleep and depression & anxiety symptoms are consistent with the overall pattern of the above results. This model illustrates that perceived physical health acts as a mediator between sleep and mental health, while school adjustment mediates the relationship between the perception of physical health and mental health. Previous studies have supported the finding that school adaption negatively associated with mental health problems [48]. In other words, the chain-mediating effect of perceived physical health and school adjustment suggests that adolescents with a lack of sleep would report that they have a poor perception of health conditions, which may influence their daily emotions and behaviors, as a result affecting school adaption, eventually end with mental health problems. Meanwhile, the study also found that insufficient sleep has no direct impact on school adjustment, which is probably because the physical changes brought by sleep deficiency are still within the range of students’ daily adapting ability. Therefore, there is no significant impact from sleep restriction but affects school adjustment in the way of psychological perception.

This study highlights the importance of focusing on improving adolescents’ mental health by increasing sleep duration, improving their perception of physical health, and school adjustment. This study may be able to inform future practices because little is known about the relationship between perceived physical health and school adjustment with sleep and mental health, as well as interactions between them. Therefore, the results of this study can be used to guide policies and strategies to improve adolescents’ mental health. For example, educational administrators can advocate delaying school start times resulting in longer weeknight sleep and producing cascading positive effects [49]. In addition, cognitive-behavioral therapy (CBT) can be used in adolescents who have struggled with poor self-rated health conditions [50]. Furthermore, students can be given full support, especially social support to improve their school adjustment, for reaching a state of mental well-being.

### Strengths and limitations

The study has several strengths and limitations that point to directions for future research. One strength of our study is that we included a large sample size of high school students and analyzed their mental health status and sleep condition at present. Another strength is that this study identified the mediators that explain how sleep influences mental health. Future studies aiming at intervening in adolescents’ mental health could consider the effects of sleep outcomes and provide a new method to improve adolescents’ mental health.

Despite its strengths, this study has some limitations that need to be acknowledged. To begin with, it had a cross-sectional design, which does not allow for establishing the causality of mediation. Research suggests that the relationship between sleep and mental health is complex. While lack of sleep has long been known to be a consequence of many psychiatric conditions [51], more recent views suggest insufficient sleep can also play a causal role in both the development and maintenance of different mental health problems [40, 52, 53]. Although we are aware that the database we used conducted several longitudinal surveys at different time points. To our knowledge, this database only published data on mental health for the semester 2020/2021. Hence, longitudinal studies should be conducted using the factors of this study to determine how and why the factors change over time. Second, due to the limitations of database sampling, the measurement of independent variable sleep mainly depends on the self-report sleep duration, which is difficult to comprehensively reflect the sleep conditions in real life. Therefore, varieties of scientific sleep measurement indexes can be considered in future research, like actigraphy and polysomnography (PSG). Finally, our study has focused on the influence of school adjustment on mental health status. However, adolescents’ school adjustment develops dynamically with different characteristics and rules in different stages, such as the transition period into senior high school and the exam preparation period. Students in these stages often produce more prominent adaption problems, therefore, the effects of school adjustment can be further explored.

## Conclusion

Our study concluded that the mental health of high school students is closely related to sleep, perception of physical health, and school adjustment. Our research findings indicated that promoting adequate sleep duration and improving sleep quality has a positive effect on building a good perception of physical health and enhancing school adjustment, which may be a key strategy for promoting mental health, especially depression and anxiety, in adolescents.

### Electronic supplementary material

Below is the link to the electronic supplementary material.

Supplementary Material 1

## Data Availability

Data were drawn from the Database of Youth Health (2021) from National Population Health Data Center (https://www.ncmi.cn/phda/dataDetails.do?id=CSTR:17970.11.A0031.202107.209.V1.0). The dataset provides information on Chinese youth on physical fitness, social interaction, nutrition and diet, school adaption, quality of life, psychological cognition, mental health, spare-time physical activity, and risk behavior. The dataset was a muti-wave survey conducted annually in the academic year of 2015/2016, 2016/2017, 2017/2018, and 2020/2021.

## References

[1] Tarokh L, Saletin JM, Carskadon MA (2016). Sleep in adolescence: physiology, cognition and mental health. Neurosci Biobehav Rev.

[2] Jenni OG, Achermann P, Carskadon MA (2005). Homeostatic sleep regulation in adolescents. Sleep.

[3] Yushu Z (2023). Sleep status among children and adolescents aged 6–17 years — China, 2016–2017. China CDC Wkly.

[4] Liang M (2021). Prevalence of sleep disturbances in Chinese adolescents: a systematic review and meta-analysis. PLoS ONE.

[5] Chen T (2014). Sleep duration in Chinese adolescents: biological, environmental, and behavioral predictors. Sleep Med.

[6] Lo JC et al. Sustained benefits of delaying school start time on adolescent sleep and well-being. Sleep, 2018. 41(6). 10.1093/sleep/zsy052 PMC5995199 29648616

[7] Medic G, Wille M, Hemels ME (2017). Short- and long-term health consequences of sleep disruption. Nat Sci Sleep.

[8] Saghir Z (2018). The Amygdala, Sleep Debt, Sleep Deprivation, and the emotion of anger: a possible connection?. Cureus.

[9] Krause AJ (2017). The sleep-deprived human brain. Nat Rev Neurosci.

[10] Palmer CA, Alfano CA (2017). Sleep and emotion regulation: an organizing, integrative review. Sleep Med Rev.

[11] Tempesta D (2018). Sleep and emotional processing. Sleep Med Rev.

[12] Deschenes SS (2019). Depressive symptoms and sleep problems as risk factors for heart disease: a prospective community study. Epidemiol Psychiatr Sci.

[13] Baum KT (2014). Sleep restriction worsens mood and emotion regulation in adolescents. J Child Psychol Psychiatry.

[14] Palagini L (2022). Sleep, insomnia and mental health. J Sleep Res.

[15] Wang Z (2022). The microbiota-gut-brain axis in sleep disorders. Sleep Med Rev.

[16] Shanahan L (2014). Sleep problems predict and are predicted by generalized anxiety/depression and oppositional defiant disorder. J Am Acad Child Adolesc Psychiatry.

[17] Chellappa SL, Aeschbach D (2022). Sleep and anxiety: from mechanisms to interventions. Sleep Med Rev.

[18] Palagini L, Rosenlicht N (2011). Sleep, dreaming, and mental health: a review of historical and neurobiological perspectives. Sleep Med Rev.

[19] Horne JA (1993). Human sleep, sleep loss and behaviour. Implications for the prefrontal cortex and psychiatric disorder. Br J Psychiatry.

[20] Foley JE, Weinraub M (2017). Sleep, affect, and social competence from Preschool to Preadolescence: distinct pathways to Emotional and Social Adjustment for boys and for girls. Front Psychol.

[21] Henriquez-Tejo R, Cartes-Velasquez R (2018). Psychosocial impact of type 1 diabetes mellitus in children, adolescents and their families. Literature review]. Rev Chil Pediatr.

[22] Coles ME (2016). Adolescent Mental Health Literacy: Young people’s knowledge of depression and social anxiety disorder. J Adolesc Health.

[23] Vie TL (2019). Self-rated health (SRH) in young people and causes of death and mortality in young adulthood. A prospective registry-based Norwegian HUNT-study. SSM Popul Health.

[24] Mikolajczyk RT (2008). Factors associated with self-rated health status in university students: a cross-sectional study in three European countries. BMC Public Health.

[25] Hossain S (2020). Self-perception of physical health conditions and its association with depression and anxiety among Bangladeshi university students. J Affect Disord.

[26] Chen Y et al. Social identity, core Self-Evaluation, School Adaptation, and Mental Health problems in migrant children in China: a chain mediation model. Int J Environ Res Public Health, 2022. 19(24). 10.3390/ijerph192416645 PMC9778830 36554527

[27] Ladd GW, Burgess KB (2001). Do relational risks and protective factors moderate the linkages between childhood aggression and early psychological and school adjustment?. Child Dev.

[28] Chui RCF, Chan C-K, Adjustment S (2017). Social Support, and Mental Health of Mainland Chinese College Students in Hong Kong. J Coll Student Dev.

[29] Steinmayr R (2015). Subjective Well-Being, test anxiety, academic achievement: testing for reciprocal effects. Front Psychol.

[30] Irwin MR (2015). Why sleep is important for health: a psychoneuroimmunology perspective. Annu Rev Psychol.

[31] Owens J, Adolescent Sleep G, Working (2014). Committee on, *Insufficient sleep in adolescents and young adults: an update on causes and consequences*. Pediatrics.

[32] Marconato RS, Monteiro MI (2015). Pain, health perception and sleep: impact on the quality of life of firefighters/rescue professionals. Rev Lat Am Enfermagem.

[33] Azpiazu L, et al. School adjustment in adolescence explained by social support, resilience and positive affect. European Journal of Psychology of Education; 2024.

[34] Vaz S (2015). Should schools expect poor physical and mental health, social adjustment, and participation outcomes in students with disability?. PLoS ONE.

[35] Garcia-Real TJ, et al. [Relationship among sleep quality, sleep habits and school adjustment in adolescents from an urban district of Galicia]. Rev Esp Salud Publica; 2020. p. 94.33223516

[36] Walker MP (2009). The role of sleep in cognition and emotion. Ann N Y Acad Sci.

[37] Zhang SF (2022). A dataset on the Status Quo of Health and Health-related behaviors of Chinese Youth: a longitudinal large-scale survey in the secondary School students of Shandong Province. Chin Med Sci J.

[38] Andrade ALM et al. The Effect of Psychosocial interventions for reducing co-occurring symptoms of depression and anxiety in individuals with problematic internet use: a systematic review and Meta-analysis. Int J Ment Health Addict, 2022: p. 1–22. 10.1007/s11469-022-00846-6 PMC9164571 35677712

[39] Cheng W (2021). Sleep duration, brain structure, and psychiatric and cognitive problems in children. Mol Psychiatry.

[40] Scott AJ, Webb TL, Rowse G (2017). Does improving sleep lead to better mental health? A protocol for a meta-analytic review of randomised controlled trials. BMJ Open.

[41] Khurshid KA (2018). Comorbid Insomnia and Psychiatric disorders: an update. Innov Clin Neurosci.

[42] Waters F (2018). Severe sleep deprivation causes hallucinations and a gradual progression toward psychosis with increasing Time Awake. Front Psychiatry.

[43] Bernert RA (2015). Sleep disturbances as an evidence-based suicide risk factor. Curr Psychiatry Rep.

[44] Buxton OM (2015). Sleep in the modern family: protective family routines for child and adolescent sleep. Sleep Health.

[45] Othman N (2019). Perceived impact of contextual determinants on depression, anxiety and stress: a survey with university students. Int J Ment Health Syst.

[46] Hilger-Kolb J (2018). Effort-reward imbalance among students at German universities: associations with self-rated health and mental health. Int Arch Occup Environ Health.

[47] El Ansari W, Ssewanyana D, Stock C (2018). Behavioral health risk profiles of Undergraduate University students in England, Wales, and Northern Ireland: a cluster analysis. Front Public Health.

[48] Kor A (2019). A longitudinal study of spirituality, character strengths, Subjective Well-Being, and Prosociality in Middle School adolescents. Front Psychol.

[49] Wheaton AG, Chapman DP, Croft JB (2016). School Start Times, Sleep, behavioral, Health, and academic outcomes: a review of the literature. J Sch Health.

[50] Freeman D (2017). The effects of improving sleep on mental health (OASIS): a randomised controlled trial with mediation analysis. Lancet Psychiatry.

[51] Nutt D, Wilson S, Paterson L (2008). Sleep disorders as core symptoms of depression. Dialogues Clin Neurosci.

[52] Baglioni C (2016). Sleep and mental disorders: a meta-analysis of polysomnographic research. Psychol Bull.

[53] Bishop TM (2016). Sleep and suicide in older adults: an opportunity for intervention. Clin Ther.

