# Supplementary material for: Sleep and mental health among Chinese adolescents: the chain-mediating role of physical health perception and school adjustment
Source: BMC Psychol. 2024 Apr 24;12:228. doi: 10.1186/s40359-024-01719-4 (PMC11044529; doi:10.1186/s40359-024-01719-4)
Supplement: Supplementary file 1 — Supplementary Material 1 [file 40359_2024_1719_MOESM1_ESM.docx]

Supplemental Table 1. School Social Behavior Scales (SSBS-2)

| No. | Subject | Options |
| --- | --- | --- |
| 1 | is cooperative with other students |  |
| 2 | can switch between various activities smoothly | circle 1 if NEVER HAPPEN; circle 2 if RARELY HAPPEN; circle 3 if MODERATE; circle 4 if OCCURS OCCASIONALLY; circle 5 if OFTEN HAPPEN1=never happen, 2=mild, 3=moderate, 4=fairly severe, and 5=severe |
| 3 | is no need to rush to get homework done from his/her seat |  |
| 4 | takes the initiative to help students |  |
| 5 | participates effectively in discuss and activities |  |
| 6 | understands the needs and questionss of other students |  |
| 7 | can stay calm when something goes wrong |  |
| 8 | listens to teacher and do what teacher says |  |
| 9 | is active to invite other students |  |
| 10 | asks teacher questions in an appropriate manner |  |
| 11 | can be worshiped by students |  |
| 12 | is accepted and liked by other students |  |
| 13 | completes assignments or tasks independently |  |
| 14 | completes assignments or tasks on time |  |
| 15 | can aggree with students |  |
| 16 | observes discipline |  |
| 17 | behaves well in many school situations |  |
| 18 | asks for help appropriately |  |
| 19 | socializes with many peers |  |
| 20 | meets others expectations |  |
| 21 | communicates with students or join in exchanges skillfully |  |
| 22 | can detect the psychological changes of other students |  |
| 23 | responds appropriately when corrected by the teacher |  |
| 24 | will not lose his/her temper when angry or enraged |  |
| 25 | incorporates activities that students are doing |  |
| 26 | well done organizational skills |  |
| 27 | adapts to the school's requirements and expectations |  |
| 28 | praises others |  |
| 29 | is decisive when needed |  |
| 30 | is elected by classmates to participate in activities |  |
| 31 | has self-control and self-restraint |  |
| 32 | is respected by peers |  |
| 33 | blames other students for prblems |  |
| 34 | take things that are not his/her |  |
| 35 | defies teacher or other school personnel |  |
| 36 | cheats on schoolwork or in games |  |
| 37 | get into fights |  |
| 38 | lies to teacher or other school personnel |  |
| 39 | tears and make fun of others students |  |
| 40 | is disrespectful or "sassy" |  |
| 41 | is easily provoked;has a short fuse |  |
| 42 | ignores teacher or other school personnel |  |
| 43 | acts as if he/she is better than thers |  |
| 44 | destroys or damages school propetty |  |
| 45 | will not share with other students |  |
| 46 | has temper outburst or tantrums |  |
| 47 | disregards feelings and needs of other students |  |
| 48 | is overly demanding of teacher's attention |  |
| 49 | threatens others students, is verballu aggressive |  |
| 50 | swears or uses obscene language |  |
| 51 | is physically aggressive |  |
| 52 | insults peers |  |
| 53 | whines and complains |  |
| 54 | argues and quarrels with peers |  |
| 55 | is difficult to control |  |
| 56 | bothers and annoys other students |  |
| 57 | get in trouble at school |  |
| 58 | disrupts ongoing actives |  |
| 59 | disrupts ongoing actives |  |
| 60 | cannot be depended on |  |
| 61 | is bad to other student |  |
| 62 | acts impulsively or without thinking |  |
| 63 | unproductive, achieves very little |  |
| 64 | is easilly irratated |  |
| 65 | is forcing other classmates to help |  |

Supplemental Table 2. Symptom Checklist 90 (SCL-90)

| No. | Subject | Options |
| --- | --- | --- |
| 1 | Headaches | 1=never, 2=mild, 3=moderate, 4=fairly severe, and 5=severe |
| 2 | Nervousness or shakiness inside |  |
| 3 | Unwanted thoughts, words, or ideas that won’t leave your mind |  |
| 4 | Faintness or dizziness |  |
| 5 | Loss of sexual interest or pleasure |  |
| 6 | Feeling critical of others |  |
| 7 | The idea that someone else can control your thoughts |  |
| 8 | Feeling others are to blame for most of your troubles |  |
| 9 | Trouble remembering things |  |
| 10 | Worried about sloppiness or carelessness |  |
| 11 | Feeling easily annoyed or irritated |  |
| 12 | Pains in heart or chest |  |
| 13 | Feeling afraid in open spaces or on the streets |  |
| 14 | Feeling low in energy or slowed down |  |
| 15 | Thoughts of ending your life |  |
| 16 | Hearing voices that other people do not hear |  |
| 17 | Trembling |  |
| 18 | Feeling that most people cannot be trusted |  |
| 19 | Poor appetite |  |
| 20 | Crying easily |  |
| 21 | Feeling shy or uneasy with the opposite sex |  |
| 22 | Feeling of being trapped or caught |  |
| 23 | Suddenly scared for no reason |  |
| 24 | Temper outbursts that you could not control |  |
| 25 | Feeling afraid to go out of your house alone |  |
| 26 | Blaming yourself for things |  |
| 27 | Pains in lower back |  |
| 28 | Feeling blocked in getting things done |  |
| 29 | Feeling lonely |  |
| 30 | Feeling blue |  |
| 31 | Worrying too much about things |  |
| 32 | Feeling no interest in things |  |
| 33 | Feeling fearful |  |
| 34 | Your feelings being easily hurt |  |
| 35 | Other people being aware of your private thoughts |  |
| 36 | Feeling others do not understand you or are unsympathetic |  |
| 37 | Feeling that people are unfriendly or dislike you |  |
| 38 | Having to do things very slowly to insure correctness |  |
| 39 | Heart pounding or racing |  |
| 40 | Nausea or upset stomach |  |
| 41 | Feeling inferior to others |  |
| 42 | Soreness of your muscles |  |
| 43 | Feeling that you are watched or talked about by others |  |
| 44 | Trouble falling asleep |  |
| 45 | Having to check and double-check what you do |  |
| 46 | Difficulty making decisions |  |
| 47 | Feeling afraid to travel on buses, subways, trains |  |
| 48 | Trouble getting your breath |  |
| 49 | Hot or cold spells |  |
| 50 | Having to avoid certain things, places, or activities because they frighten you |  |
| 51 | Your mind going blank |  |
| 52 | Numbness or tingling in parts of your body |  |
| 53 | A lump in your throat |  |
| 54 | Feeling hopeless about the future |  |
| 55 | Trouble concentrating |  |
| 56 | Feeling weak in parts of your body |  |
| 57 | Feeling tense or keyed up |  |
| 58 | Heavy feelings in your arms or legs |  |
| 59 | Thoughts of death or dying |  |
| 60 | Overeating |  |
| 61 | Feeling uneasy when people are watching or talking about you |  |
| 62 | Having thoughts that are not your own |  |
| 63 | Having urges to beat, injure, or harm someone |  |
| 64 | Awakening in the early morning |  |
| 65 | Having to repeat the same actions such as touching, counting, washing |  |
| 66 | Sleep that is restless or disturbed |  |
| 67 | Sleep that is restless or disturbed |  |
| 68 | Having ideas or beliefs that others do not share |  |
| 69 | Feeling very self-conscious with others |  |
| 70 | Feeling uneasy in crowds, such as shopping or at a movie |  |
| 71 | Feeling everything is an effort |  |
| 72 | Spells of terror or panic |  |
| 73 | Feeling uncomfortable about eating or drinking in public |  |
| 74 | Getting into frequent arguments |  |
| 75 | Feeling nervous when you are left alone |  |
| 76 | Others not giving you proper credit for your achievements |  |
| 77 | Feeling lonely even when you are with people |  |
| 78 | Feeling so restless you couldn’t sit still |  |
| 79 | Feelings of worthlessness |  |
| 80 | Feeling that familiar things are strange or unreal |  |
| 81 | Shouting or throwing things |  |
| 82 | Feeling afraid you will faint in public |  |
| 83 | Feeling that people will take advantage of you if you let them |  |
| 84 | Having thoughts about sex that bother you a lot |  |
| 85 | The idea that you should be punished for your sins |  |
| 86 | Feeling pushed to get things done |  |
| 87 | The idea that something serious is wrong with your body |  |
| 88 | Never feeling close to another person |  |
| 89 | Feelings of guilt |  |
| 90 | The idea that something is wrong with your mind |  |

Supplemental Table 3. Mediating effect analysis between sleep restriction and depressive symptoms and anxiety

| **Items** | **Effect size** | **Boot SE** | **Boot CI** | | **The Proportion of Effect Size** |
| --- | --- | --- | --- | --- | --- |
|  |  |  | **Lower** | **Upper** |  |
| **Sleep restriction and depressive symptoms** | | | | |  |
| Total indirect effects | -0.044 | 0.004 | -0.052 | -0.036 | 35.74% |
| Indirect effect 1 | -0.038 | 0.003 | -0.043 | -0.033 | 31.07% |
| Indirect effect 2 | -0.011 | 0.001 | -0.013 | -0.009 | 8.85% |
| Indirect effect 3 | 0.005 | 0.003 | -0.001 | 0.011 |  |
| **Sleep restriction and anxiety** | | | | |  |
| Total indirect effects | -0.039 | 0.004 | -0.046 | -0.031 | 35.67% |
| Indirect effect 1 | -0.034 | 0.003 | -0.039 | -0.029 | 30.96% |
| Indirect effect 2 | -0.010 | 0.001 | -0.012 | -0.008 | 8.96% |
| Indirect effect 3 | 0.005 | 0.003 | -0.001 | 0.010 |  |

Note: In the model of sleep restriction and depressive symptoms, Indirect effect 1: sleep duration – perception of physical health – depression; Indirect effect 2: sleep duration – perception of physical health – school adjustment –depression; Indirect effect 3: sleep duration – school adjustment – depression.

In the model of sleep restriction and anxiety, Indirect effect 1: sleep duration – perception of physical health – anxiety; Indirect effect 2: sleep duration – perception of physical health – school adjustment –anxiety; Indirect effect 3: sleep duration – school adjustment – anxiety.
